# Although Anatomically Micrometers Apart: Human Periodontal Ligament Cells Are Slightly More Active in Bone Remodeling Than Alveolar Bone Derived Cells

**DOI:** 10.3389/fcell.2021.709408

**Published:** 2021-09-20

**Authors:** Rebecca Loo-Kirana, Marjolijn Gilijamse, Jolanda Hogervorst, Ton Schoenmaker, Teun J. de Vries

**Affiliations:** ^1^Department of Periodontology, Academic Centre For Dentistry Amsterdam, University of Amsterdam and Vrije Universiteit Amsterdam, Amsterdam, Netherlands; ^2^Department of Oral and Maxillofacial Surgery and Oral Pathology, Amsterdam UMC, Location VUmc, Amsterdam, Netherlands; ^3^Department of Oral and Maxillofacial Surgery, Onze Lieve Vrouwe Gasthuis, Amsterdam, Netherlands; ^4^Department of Oral Cell Biology, Academic Centre For Dentistry Amsterdam, University of Amsterdam and Vrije Universiteit Amsterdam, Amsterdam, Netherlands

**Keywords:** periodontal ligament cells, alveolar bone, bone remodeling, osteogenesis, osteoclastogenesis, human

## Abstract

The periodontal ligament (PDL) and the alveolar bone are part of the periodontium, a complex structure that supports the teeth. The alveolar bone is continuously remodeled and is greatly affected by several complex oral events, like tooth extraction, orthodontic movement, and periodontitis. Until now, the role of PDL cells in terms of osteogenesis and osteoclastogenesis has been widely studied, whereas surprisingly little is known about the bone remodeling capacity of alveolar bone. Therefore, the purpose of this study was to compare the biological character of human alveolar bone cells and PDL cells in terms of osteogenesis and osteoclastogenesis *in vitro*. Paired samples of PDL cells and alveolar bone cells from seven patients with compromised general and oral health were collected and cultured. Bone A (early outgrowth) and bone B (late outgrowth) were included. PDL, bone A, bone B cell cultures all had a fibroblast appearance with similar expression pattern of six mesenchymal markers. These cultures were subjected to osteogenesis and osteoclastogenesis assays. For osteoclastogenesis assays, the cells were co-cultured with peripheral blood mononuclear cells, a source for osteoclast precursor cells. The total duration of the experiments was 21 days. Osteogenesis was slightly favored for PDL compared to bone A and B as shown by stronger Alizarin red staining and higher expression of RUNX2 and Collagen I at day 7 and for ALP at day 21. PDL induced approximately two times more osteoclasts than alveolar bone cells. In line with these findings was the higher expression of cell fusion marker DC-STAMP in PDL-PBMC co-cultures compared to bone B at day 21. In conclusion, alveolar bone contains remodeling activity, but to a different extent compared to PDL cells. We showed that human alveolar bone cells can be used as an *in vitro* model to study bone remodeling.

## Introduction

The periodontal ligament (PDL) and the alveolar bone are part of the periodontium, a complex structure that supports the teeth. The alveolar bone is the part of the jawbone that contains the sockets for the teeth and in essence forms in relation to teeth (Saffar et al., 1997; Sodek and McKee, 2000). The alveolar bone is linked to the root surface by collagen fibers of the PDL, an approximately 100–400 μm thin soft and specialized connective tissue. Its collagen fibers stretch out from the root cementum into the alveolar bone. The fibroblast is the predominant cell type of the PDL, which further contains epithelial cells and undifferentiated mesenchymal stem cells (Beertsen et al., 1997).

The alveolar bone is continuously remodeled (Saffar et al., 1997; Sodek and McKee, 2000) and is greatly affected by several complex intra-oral events, three of which are as follows. Firstly, after tooth extraction, the alveolar bone is substantially remodeled, resulting in a loss of its dimension but with bone fill in the socket to a certain degree (Cardaropoli et al., 2003). Secondly, during orthodontic tooth movement, bone apposition occurs at the tension side of the tooth, during which PDL fibers are stretched, and bone is resorbed at the pressure side, where compression of the PDL fibers takes place (Feller et al., 2015). Finally, in case of periodontitis, a plaque-associated chronic multifactorial inflammatory disease, the alveolar bone is progressively resorbed and eventually can lead to tooth loss (Papapanou et al., 2018). These three examples demonstrate that knowledge of the processes that underlie the resorption and apposition of alveolar bone is of high relevance for understanding the biological basis of these common clinical events.

Bone remodeling consists of two tightly controlled processes—bone formation by osteoblasts and bone degradation by osteoclasts (Crockett et al., 2011). Osteoblasts and osteoclasts originate from different sources. Osteoblasts arise from mesenchymal stem cells (Blair et al., 2017), whereas multinucleated osteoclasts originate from the monocyte/macrophage lineage of the hematopoietic stem cells in the bone marrow or from monocytes in peripheral blood (Madel et al., 2019). The formation of osteogenic cells or osteogenesis capacity of different cells *in vitro* can be studied by stimulating the cells with osteogenic medium consisting of β-glycerophosphate (Chung et al., 1992) and ascorbic acid (Franceschi et al., 1994). Formation of osteoclasts, or osteoclastogenesis can be stimulated *in vitro* by co-culturing the cells with peripheral blood mononuclear cells (PBMCs). The differentiation of monocytes within the PBMCs into osteoclasts is achieved by the signals that are provided by the cells they are co-cultured with, for example the PDL fibroblasts (de Vries et al., 2006).

Until now, the role of PDL cells on the differentiation into osteoblast-like cells and on the osteoclastogenesis supporting role has been widely studied *in vitro*. These cells can contribute to both processes (de Vries et al., 2018). Human PDL cells have phenotypes characteristics of osteoblast-like cells (Basdra and Komposch, 1997) and can initiate mineral-like nodules *in vitro* (Arceo et al., 1991). PDL fibroblasts also have the capacity to attract osteoclast precursors and enable migration of these cells to the bone surface, giving rise to osteoclasts (Bloemen et al., 2010). *In vivo* mouse models, with for instance tooth extraction models, further support an important role for stem cells derived from the PDL in bone formation (Yuan et al., 2018). Whereas the bone remodeling capacity of PDL is well documented, surprisingly little is known about the bone remodeling capacity of alveolar bone derived cells, which are anatomically within micrometer range of the PDL cells. The alveolar bone is continuously remodeled and it is therefore plausible that its cells play an essential role in both osteogenesis and osteoclastogenesis. Alveolar bone cells from adult rabbits showed the ability to form mineralized tissue nodules with bone characteristics (Suzuki et al., 1993). This formation of mineral nodules by human alveolar bone cells has also been confirmed (Cabral et al., 2007). Human alveolar bone also contains stem cells with osteogenic potential (Matsubara et al., 2005). Sodek and McKee (2000) reviewed the biology of human alveolar, but due to the lack of data, the information included was extrapolated from studies of other human bone tissues. The authors extrapolated similarity in composition of the extracellular matrix between alveolar bone and other bone tissues to commonly shared osteogenic functions. Only recently we showed that alveolar bone cells and long bone cells may have a different morphology once cultured *in vitro*, indicating that cells from the two different types of bones may differ in their osteogenic and osteoclastogenic capacity (Kelder et al., 2020).

While PDL cells can be readily obtained by scraping off cells of the middle one third of the root surface of an extracted tooth and subsequently cultured for research purposes (Basdra and Komposch, 1997), collection of alveolar bone requires an extra step in addition to tooth extraction. The emphasis on PDL cells could potentially flaw our understanding of the bone remodeling dynamics of the periodontium, since an important feature of PDL is the maintenance of its width over time (PDL homeostasis). The biological function of PDL cells is hence to prevent osteoclast formation at the root surface (Sokos et al., 2015) and to prevent bone formation to avoid tooth ankylosis, where the tooth is fused to the alveolar bone. Although the pathogenesis of tooth ankyloses is not fully understood, cells from the root cementum and the alveolar bone play important roles since the PDL is often damaged and necrotic at sites of ankylosis (Wu et al., 2019).

Periodontal ligament cells are currently regarded as the golden standard in bone remodeling research in dentistry. Since its above mentioned biological function and given its anatomically location within micrometers range of alveolar bone, it is of high interest to compare the bone remodeling capacity of alveolar bone to the capacity of PDL cells to further understand this complex process. Ruppeka-Rupeika et al. (2018) were the first, in a case study, to describe both the bone formation and contribution to osteoclast formation of alveolar bone derived cells by comparing them with PDL cells. For the present study, we sampled PDL cells and alveolar bone cells from seven patients, where alveolar bone was well defined, as interdental alveolar crest bone chips. This enabled a pairwise comparison between the ability of PDL cells and alveolar bone cells to contribute to osteogenesis and osteoclastogenesis *in vitro*. Given the alleged susceptibility of bone turnover of alveolar bone and the biologically protective function of PDL, we hypothesized that the bone remodeling capacity of alveolar bone derived cells is higher than the capacity of PDL cells.

## Materials and Methods

### Patient Selection

Cells were derived from human interdental alveolar bone and from the adjacent tooth root surface. Patients who were referred for multiple adjacent tooth extractions and immediate removable denture placement were asked to participate. Interdental alveolar bone crest was regarded as surgical waste, since this bony rim had to be smoothened after tooth extraction to ensure a better fit of the denture. All treatments took place at the department of Oral and Maxillofacial Surgery at OLVG Amsterdam, Netherlands. Patients suffering from any blood borne systemic disease (human immunodeficiency virus, hepatitis B and C) were excluded. No other exclusion criteria were used. All patients received verbal and written information about the purpose of the study at intake. Prior to the treatment, a written informed consent was signed and a Dutch questionnaire to obtain information their general health, including smoking habits, and demographic information was filled in. The study protocol was approved by the Research Ethics Committee of OLVG (protocol-ID: WO 17.194).

### Sample Collection

A panoramic radiograph was taken to assess the root morphology of the teeth and to assess the alveolar bone height prior to tooth extraction. All patients were treated by the same maxillofacial surgeon (MG) under general anesthesia. The teeth were extracted as atraumatic as possible using forceps and elevators. To support the denture as evenly as possible, the sharp part of the interdental alveolar bone was resected using a bone rongeur. From each patient, one tooth and the resected alveolar bone chips were put in separate 50 mL tubes containing Dulbecco’s Modified Eagle Medium (DMEM, Gibco BRL, Paisley, United Kingdom) and 2% antibiotics (penicillin, streptomycin, fungizone (PSF); Sigma-Aldrich, Saint Louis, MO, United States). The tubes were stored at 4°C until transfer to the laboratory on the same day. In total, seven of these paired samples of PDL and alveolar bone outgrowths were included.

### Cell Cultures

The samples were transported within 24 h from OLVG to the department of Periodontology of the Academic Centre for Dentistry Amsterdam (ACTA), The Netherlands. The researchers of ACTA (TdV, RL-K) obtained the information from the filled in questionnaires, but the patients’ identity was kept anonymous. These files were stored at OLVG (MG). Bone chips of the alveolar bone were cut into smaller fragments of approximately 1 mm by 1 mm. These fragments underwent a collagenase (Collagenase II, Worthington, Lakewood, NJ, United States) treatment (2 mg/ml in DMEM) for 2 h to remove superficial connective tissue, enabling outgrowth of bone cells. PDL was scraped off the mid-third of the root length from the extracted tooth. PDL cells and alveolar bone cells (bone A) were cultured in a six-well plate at 37°C. The culture medium consisted of DMEM, 10% FetalClone I serum (FCI, HyClone, Logan, UT, United States) and 1% PSF. The medium was replaced every 3–4 days. Upon confluence, the cells were transferred from the well plate to a 75 cm^2^ flask (passage 1). They were further transferred to a 175 cm^2^ flask (passage 2) and trypsinized. When the six wells of bone fragments were trypsinized for the first time, these fragments were transferred to a fresh six-well plate and new outgrowth (bone B) was passaged as described for Bone A. Bone B) represents bone cells that grew out later from the bone chip. PDL cells, bone A and bone B from each patient were stored in liquid nitrogen until preparation of the cells for the osteogenesis and osteoclastogenesis assays. Apart from one sample, all PDL, Bone A and Bone B samples were successfully stored, *n* = 14 was frozen in the liquid nitrogen. For the experiments, paired samples PDL, bone A and bone B were randomly chosen. All experiments were performed with cells of passage 5.

### Osteogenesis

The three different cell cultures (PDL cells, bone A, and bone B) were seeded in duplicate in 48-well plates (3.0 × 10^4^ cells/well). Ascorbic acid at 50 μg/mL (Sigma-Aldrich) and 10 nM β-glycerophosphatase (Sigma-Aldrich), both conducive to mineralization, were added to the culture medium (see cell cultures). This mineralization medium (0.4 mL per well) was replaced every 3–4 days. The total duration of the experiment was 21 days. The osteogenesis assays included measurement of alkaline phosphatase (ALP) activity, measurement of calcium concentration, Alizarin red staining and assessment of several osteogenesis markers with quantitative polymerase chain reaction (qPCR) at different time points.

#### DNA Concentration and Alkaline Phosphatase Activity

Cells were harvested at day 0, 3, 7, 14, and 21 of culturing. In order not to sample dead cells, the adherent cells were washed twice with PBS. Next, they were lysed in 150 μL MilliQ per well and stored in -20°C. Prior to analysis, the plates underwent three cycles of freeze-thawing. ALP activity was measured using 4-nitrophenyl phosphate disodium salt (Merck, Darmstadt, Germany) at pH 10.3 as a substrate for ALP according to the method described by Bastidas-Coral (Bastidas-Coral et al., 2016). After incubation of 60 min at 37°C, the reaction was stopped with sodium hydroxide. Absorbance was measured with 405 nm with a Synergy HT spectrophotometer (BioTek Instruments Inc., Winooski, VT, United States). DNA concentration (ng/mL) was measured using CyQuant Cell Proliferation Assay Kit (Molecular Probes, Leiden, Netherlands) mixed with lysis buffer. Fluorescence was measured at 485 nm excitation and 528 nm emission with a Synergy HT spectrophotometer (BioTek Instruments Inc., Winooski, VT, United States). ALP activity was expressed as ALP per DNA (nMol/ng).

#### Calcium Concentration

The calcium assay was performed at day 14 and day 21 according to the method described by Connerty and Briggs (1966). At both time points, the culture medium was removed and the 48-well plates were stored in –20°C until analysis. Prior to the analysis, 0.5 mL 0.5 N acetic acid was added to the residue in the plates. The working solution was composed of 14.8 M ethanolamine/boric acid buffer (pH 11), o-cresolphtalein complexone, 8-hydroxyquinoline (Sigma-Aldrich) and MilliQ. After incubation of 5-10 minutes at room temperature, the absorbance was measured at 570 nm with a Synergy HT spectrophotometer (BioTek Instruments Inc., Winooski, VT, United States). Calcium concentration was expressed in μg/mL.

#### Alizarin Red Staining

Alizarin red staining was performed after 21 days to analyze the mineral deposition. All three cell cultures were seeded in duplicate in two separate 48-well plates. Per well plate, the mineralization medium (as mentioned in 2.4) was added to one well of each cell culture. Culture medium only was added to the control well. 2% Alizarin red S at pH 4.3 (Sigma-Aldrich) was used for staining. Cells were fixed for 10 min in 4% formaldehyde and rinsed with deionized water before adding 300 μL of 2% Alizarin solution per well. After incubation of 15 min at room temperature, the cells were washed with deionized water and air-dried. Mineral deposition was visualized in red nodules.

### Osteoclastogenesis

Osteoclast formation was assessed in parallel to the osteogenesis experiment, with an equal duration of the experiment of 21 days. The cell cultures were seeded in duplicate one day in advance in a 48-well plate at 1.5 × 10^4^ cells per/well. They were co-cultured with 0.5 × 10^6^ cells/well PBMCs. The PBMCs were isolated from a buffy coat (Sanquin, Amsterdam, Netherlands), using ficoll density gradient centrifugation (GE Healthcare Bio-Sciences AB, Uppsala, Sweden) as described previously (Ruppeka-Rupeika et al., 2018). The culture medium composed of DMEM, 10% fetal clone I serum, and 1% PSF (as described in 2.3) was used. The osteoclastogenesis assays included osteoclast quantification (see 2.5.1) and assessment of several osteoclastogenesis markers with qPCR (see 2.6) at different time points.

#### Osteoclast Quantification

Osteoclast quantification was performed after 21 days of culturing. Phosphate buffered saline with 4% formaldehyde was used to fix the cells on plastic. Cells were stained for the presence of Tartrate-resistant acid phosphatase (TRAcP) using the Acid Phosphatase, Leukocyte kit (Sigma-Aldrich), following the instructions of the manufacturer. Diamidino-2-phenylindole dihydrochloride (DAPI, Life Technologies) was used to stain the nuclei. Micrographs were taken from five fixed positions per well with a digital camera (Leica, Wetzlar, Germany) and analyzed for the number of TRAcP-positive multinucleated cells. Cells were considered as multinucleated osteoclast-like cell when containing at least three nuclei.

### Quantitative Polymerase Chain Reaction

Quantitative polymerase chain reaction (qPCR) analysis was performed for mesenchymal markers, and for osteogenesis and osteoclastogenesis at day 0, 7, and 21. At these time points the culture medium was removed and 200 μL RNA lysis buffer (Qiagen, Hilden, Germany) was added per well. Subsequently, the 48-well plates were stored in -80°C until further use. RNA isolation was performed with Qiagen RNeasy Mini kit according to the manufacturer’s instructions. The RNA concentration and quality was determined using absorption read at 260 and 280 nm with Synergy HT spectrophotometer (BioTek Instruments Inc., Winooski, VT, United States). RNA was reverse transcribed to cDNA with the MBI Fermentas cDNA synthesis Kit (Vilnius, Lithuania). Oligo(dT)18 and D(N)6 were used as primers. Real time primers were designed for several genes. The included markers for osteogenesis were Dentin matrix acidic phosphoprotein 1 (DMP1), Runt-related transcription factor 2 (RUNX2), Collagen I (COLI), Osteonectin, ALP, and Sclerostin. For osteoclastogenesis, the markers included Receptor activator of nuclear factor kappa-B (RANK) and -ligand (RANKL), Osteoprotegerin (OPG), Macrophage colony-stimulating factor (M-CSF). Tartrate-resistant acid phosphatase (TRAcP) and Dendritic cell-specific transmembrane protein (DC-STAMP) were the included osteoclast markers. PCR was performed on the LC480 light cycler (Roche, Basel, Switzerland). Beta-2-microglobulin (β2M) was used as a housekeeping gene for mesenchymal markers and the osteogenesis markers, while hypoxanthine phosphoribosyltransferase 1 (HPRT1) was used as housekeeping gene for the mesenchymal markers and osteoclast markers. The value of the genes was normalized for β2M or HPRT1 expression following the comparative threshold (Ct) method. ΔC (Ct _gene of interest_ – Ct _housekeeping gene_) was calculated and relative expression of the genes was determined as 2^–ΔCt^. The primer sequences are listed in Table 1.

**TABLE 1:** Primer sequences used for quantitative polymerase chain reaction (qPCR).

**Gene**	**Sequence**	**Amplicon length**	**Ensembl gene ID**
**Mesenchymal markers**			
*PLAP-1*	5′ TCACTTTATggTCTgATCCTgAACA 3′	70	ENSG00000106819
	5′ TTTgTggTTAgAAAggCTTTTgg 3′		
*FAPα*	5′ AgCgACTACgCCAAgTACTATgC 3′	69	ENSG00000078098
	5′ CATCATgAAgggTggAAATgg 3′		
*Periostin*	5′ CCCAgCAgTTTTgCCCATT 3′	60	ENSG00000133110
	5′ TgTggTggCTCCCACgAT 3′		
*Scleraxis*	5′ gAACACCCAgCCCAAACAgAT 3′	62	ENSG00000260428
	5′ TCCTTgCTCAACTTTCTCTggTT 3′		
*CD73*	5′ ggAggACACTCCAACACATT 3′	327	ENSG00000135318
	5′ ggAgCCATCCAgATAgACAA 3′		
*Vimentin*	5′ TgCgTCTCTggCACgTCTTgA 3′	217	ENSG00000026025
	5′ CAggTTCTTggCAgCCACACT 3′		
***Osteogenesis***			
*β2M*	5′ AAGATTCAGGTTTACTCACGTC 3′	273	ENSG00000166710
	5′ TGATGCTGCTTACATGTCTCG 3′		
*DMP1*	5′ CCTCTTTGAGAACATCAACCTGATTT 3′	106	ENSG00000152592
	5′ GAGCAGGATGCTGATCTTCATAGTT 3′		
*RUNX2*	5′ ATGCTTCATCGCCTCAC 3′	156	ENSG00000124813
	5′ ACTGCTTGCAGCCTTAAAT 3′		
*COL1A1*	5′ TCCAACGAGATCGAGATCC 3′	190	ENSG00000108821
	5′ AAGCCGAATTCCTGGTCT 3′		
*Osteonectin*	5′ TACATCGGGCCTTGCAAATAC 3′	100	ENST00000231061
	5′ AGGGTGACCAGGACGTTCTTG 3′		
*ALP*	5′ GCTTCAAACCGAGATACAAGCA 3′	101	ENSG00000162551
	5′ GCTCGAAGAGACCCAATAGGTAGT 3′		
*Sclerostin*	5′ GGGTGGCAGGCGTTCA 3′	163	ENSG00000167941
	5′ CTGTACTCGGACACGTCTTTGGT 3′		
**Osteoclastogenesis**			
*HPRT*	5′ TGACCTTGATTTATTTTGCATACC 3′	101	ENSG00000165704
	5′ CGAGCAAGACGTTCAGTCCT 3′		
*RANK*	5′ CCTGGACCAACTGTACCTTCCT 3′	67	ENSG00000141655
	5′ ACCGCATCGGATTTCTCTGT 3′		
*RANKL*	5′ CATCCCATCTGGTTCCCATAA 3′	60	ENSG00000120659
	5′ GCCCAACCCCGATCATG 3′		
*M-CSF*	5′ CCGAGGAGGTGTCGGAGTAC 3′	100	ENSG00000184371
	5′ AATTTGGCACGAGGTCTCCAT 3′		
*OPG*	5′ CTGCGCGCTCGTGTTTC 3′	100	ENSG00000164761
	5′ ACAGCTGATGAGAGGTTTCTTCGT 3′		
*DC-STAMP*	5′ ATTTTCTCAGTGAGCAAGCAGTTTC 3′	101	ENSG00000164935
	5′ AGAATCATGGATAATATCTTGAGTTCCTT 3′		
*TRAcP*	5′ CACAATCTGCAGTACCTGCAAGGAT 3′	128	ENSG00000102575
	5′ CCCATAGTGGAAGCGCAGATA 3′		

**FIGURE 1:**
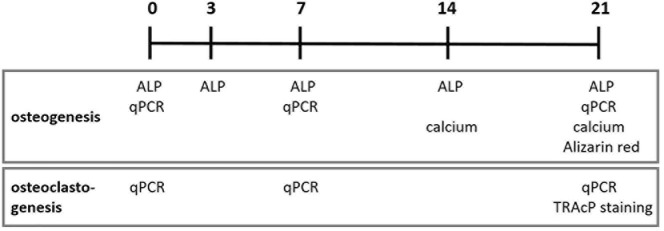
Osteogenesis and osteoclastogenesis assays at different time points in days. ALP, alkaline phosphatase activity; qPCR, quantitative polymerase chain reaction; TRAcP, Tartrate-resistant acid phosphatase.

An overview of the performed osteogenesis and osteoclastogenesis assays at several time points is shown in Figure 1.

### Statistical Analyses

Since the three cell cultures were collected from the same patient, paired comparison was made for the osteogenesis and osteoclastogenesis assays. Data are presented as mean + standard deviation (SD). When normally distributed data were at stake (*n* = 5), the differences between the three cell cultures within the several time points and the differences per cell culture over time were statistically analyzed using the non-parametric Friedman test with Dunn’s multiple comparison *post hoc* test. The Wilcoxon signed rank test was used to measure the difference between day 14 and 21 for calcium concentration. Cells from five out of seven patients were used for the quantitative osteogenesis assays, since, for two patients not enough cells to perform all experiments could be obtained. For the osteoclastogenesis assays, samples from seven patients were included and were normally distributed as confirmed by the Sapiro–Wilk Test. Therefore, One-way ANOVA (repeated measures) with a Tukey’s multiple comparison *post hoc* test was used. The significance level was set at *p* < 0.05. GraphPad Prism 5.0 (GraphPad Software, San Diego, CA, United States) was used to perform the analyses. When not normally distributed, one-way ANOVA was used with Dunn’s comparison of all columns was used as statistical test.

## Results

### Patients Characteristics

Patients characteristics were obtained from the filled in questionnaires. Primary cells from seven patients were included. In total seven paired tissue samples, each including an extracted tooth and bone chips from the alveolar crest were collected. Mean age of the patients was 51 years (range 21–75 years). An overview of the type of extracted tooth, the age and sex of the patient is shown in Table 2.

**TABLE 2:** Extracted tooth, age and sex of the patient.

**Patient**	**Age**	**Sex**	Extracted tooth*
1	67	M	37
2	60	F	47
3	21	F	28
4	41	F	44
5	57	F	44
6	75	M	42
7	37	M	44
**Mean age**	**51 (±18.9)**		

(Mean) age in years (SD). M, male; F, female. *According to the ISO-system (3950) from the World Health Organization.

### Cell Characterization

**FIGURE 2:**
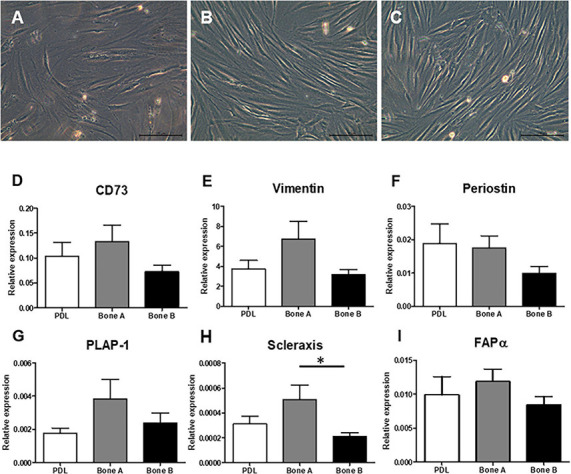
Fibroblast appearance of **(A)** PDL derived cells and alveolar bone derived cells **(B)** bone A and **(C)** bone B from patient 1. Images were taken prior to the start of the experiments. Only culture medium (DMEM) was added. These cells were not subjected to osteogenic or osteoclastogenic medium. Bar = 100 μm. Analysis of gene expression of mesenchymal markers **(D)** CD73, **(E)** Vimentin, **(F)** Periostin, **(G)** PLAP-1, **(H)** Scleraxis, and **(I)** FAPα revealed similar gene expression between the cells (*n* = 7). Expression was relative to housekeeping gene HPRT. ^∗^*p* < 0.05 (Friedman test with Dunn’s multiple comparison *post hoc* test).

Three different samples (PDL, bone A, and bone B) per patient were cultured. Microscopic images of these three cell cultures are shown in Figure 2. A fibroblastic appearance was noted for all three cell types (Figures 2A–C), microscopically no clear differences could be observed between PDL cells and the alveolar bone cells (bone A and B). To further study possible differences between the cell types, we next assessed expression of markers that are highly expressed in PDL fibroblasts and partly known to be expressed in osteoblasts as well. These were: mesenchymal marker CD73 that is expressed by all PDL fibroblasts according to Abedian et al. (Abedian et al., 2020; Figure 2D), mesenchymal cytoskeleton marker Vimentin (Figure 2E), and Periostin (Horiuchi et al., 1999; Figure 2F), PDL associated protein-1 (PLAP-1) (Yamada et al., 2007; Figure 2G), Scleraxis (Takimoto et al., 2015; Figure 2H), and fibroblast activation protein-α (FAPα) (Driesen et al., 2020; Figure 2I). Apart from a significant difference between bone A and bone B for Scleraxis, no differences between the three cell isolates were found. Together, this characterization shows that these three cell lineages have a similar appearance and a mesenchymal expression pattern.

### Osteogenesis Assays

#### Similar Proliferation for all Cell Types

**FIGURE 3:**
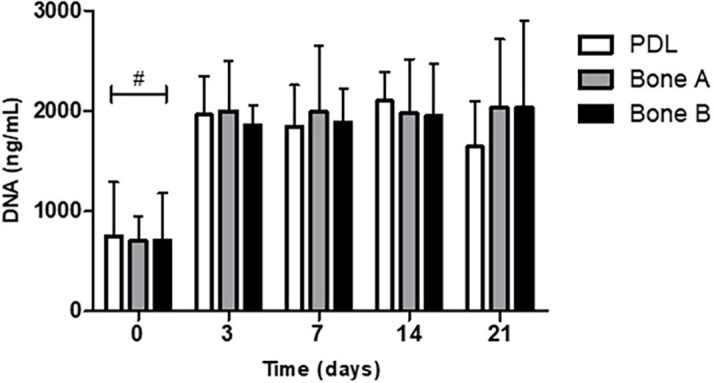
DNA concentration of PDL cells and alveolar bone cells bone A and B at day 0, 3, 7, 14, and 21. Means ± SD are shown (*n* = 5). No significant differences were found between the 3 cell cultures at all time points. ^#^*p* < 0.05 between day 0 and the other time points for PDL, bone A, bone B (Friedman test with Dunn’s multiple comparison *post hoc* test).

We first measured the DNA concentration of the different cells over time, as a measure of proliferation. DNA concentration was measured at day 0, 3, 7, 14, and 21 (Figure 3). The DNA content at day 3, 7, 14, and 21 was statistically significant higher than at day 0 for PDL, bone A and bone B. No significant differences were found between day 3, 7, 14, and 21. In addition, no significant differences were found between PDL, bone A and bone B at each time point. From these experiments, we concluded that there were no differences between the three cell types in terms of DNA concentration. Therefore, possible differences in osteogenesis later on cannot be explained by differences in cell densities.

Next, differences in osteogenic differentiation were assessed by measuring ALP enzyme activity, calcium deposition, Alizarin red staining and by assessing osteogenic markers using qPCR.

#### Alkaline Phosphatase Activity Increases Over Time for all Three Cell Cultures

**FIGURE 4:**
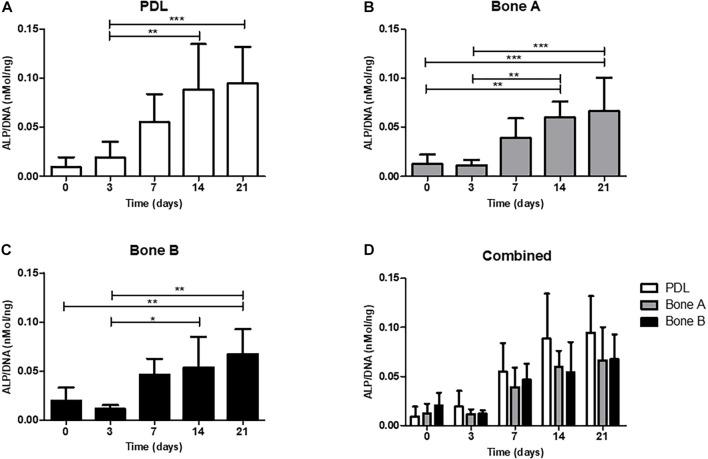
Alkaline phosphatase activity of **(A)** PDL, **(B)** bone A, **(C)** bone B, and **(D)** the three cell cultures combined. Alkaline phosphatase activity was assessed at day 0, 3, 7, 14, 21. Significant differences between time points are shown in panels **(A–C)**. No significant differences were found between PDL, bone A and b one B at each time point. Alkaline phosphatase activity is measured per cell, corrected for DNA. Means ± SD are shown (*n* = 5). *p < 0.05; **p < 0.01; ***p < 0.001 (Friedman test with Dunn’s multiple comparison *post hoc* test).

Alkaline phosphatase activity is shown in Figure 4. The activity is expressed as ALP per DNA (nMol/ng). For all three cell cultures an increase of ALP activity was observed over time and peak expression was at day 14. A statistically significant increase at day 14 (*p* < 0.01) and at day 21 (*p* < 0.001) compared to day 3 was observed for PDL (Figure 4A). The activity level from bone A was significantly higher at day 14 and 21 compared to day 0 and 3 (*p* < 0.01) (Figure 4B). For bone B, a significant increase of the ALP activity was found between day 0 and day 21 (*p* < 0.01), and between day 3 and day 14, 21 (*p* < 0.05) (Figure 4C). When compared per time point (Figure 4D), PDL seemed to show higher activity compared to bone A and bone B at day 7, 14, and 21. However, these differences were not statistically significant. In addition, no differences between bone A and B were found at any time point.

#### Similar Calcium Deposits by PDL and Alveolar Bone Cells in Osteogenic Medium

**FIGURE 5:**
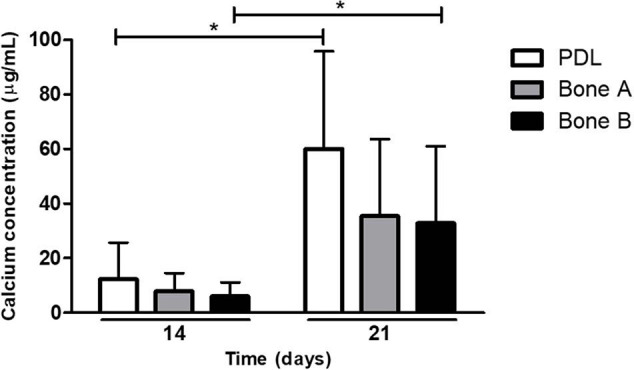
Calcium deposits by PDL, bone A and bone B at day 14 and 21. Calcium concentration of PDL and bone B were significantly higher at day 21 compared to day 14. No significant differences were found between the three cell cultures at day 14 or day 21 (Friedman test with Dunn’s multiple comparison *post hoc* test). Means ± SD are shown (*n* = 5). *p < 0.05 (Wilcoxon signed rank test).

A second way to assess osteogenesis, is the capacity to deposit calcium. Calcium deposition occurs at late time points during osteogenesis. Calcium deposits by PDL, bone A, and bone B at day 14 and day 21 is depicted in Figure 5. Although there seemed to be an increase in concentration over time for all three cell cultures, only for PDL and bone B the difference between day 14 and day 21 reached significance (*p* < 0.05). In addition, both at day 14 and day 21 the calcium concentration of PDL seemed to be higher compared to the calcium concentration of bone A and B, whereas no clear difference in concentration between bone A and B was observed. However, the calcium concentration of PDL was for both day 14 and 21 not significantly different compared to bone A and bone B.

#### PDL Shows Stronger Alizarin Red Staining Compared to Alveolar Bone Cells

**FIGURE 6:**
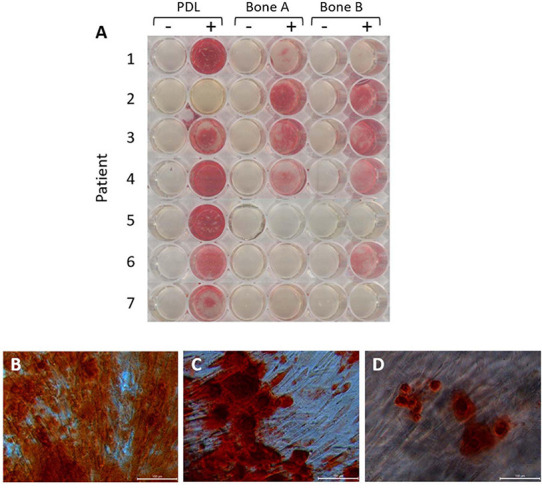
Alizarin red staining after 21 days of osteogenic culture. **(A)** Wells without osteogenic medium (–) and with osteogenic medium (+) for PDL, bone A and bone B from 7 patients. Microscopic images of **(B)** PDL+, **(C)** Bone A+, **(D)** bone B+ from patient 1. Bar = 100 μm.

Thirdly, mineral deposition can by studied with Alizarin red staining. This staining was performed after 21 days, also visualizing the nodular mineral deposition (Figure 6A). We recently demonstrated that this nodular aspects further branch into smaller nodules, possibly connected with threads that may resemble collagen fibrils, when examined by scanning electron microscopy (de Vries et al., 2019; Karlis et al., 2020). For all patients, except for patient 2, the strongest staining was observed for PDL compared to bone A and bone B. For patient 2, much stronger staining could be found for bone A and B compared to PDL. Each well did contain cells, as revealed under the microscope. Regarding the Alizarin red staining in bone A and B, more variety was shown between the patients. Apart from one case, no clear difference in staining was apparent between bone A and B. Some microscopic images of selected wells are shown in Figures 6B–D. Alizarin red staining was absent when osteogenic medium was not added. As an example, mineral deposition visualized by the staining is clearly visible in PDL (Figure 6B), bone A (Figure 6C), and B (Figure 6D) from patient 1.

#### qPCR Analysis of Osteogenesis Genes

**FIGURE 7:**
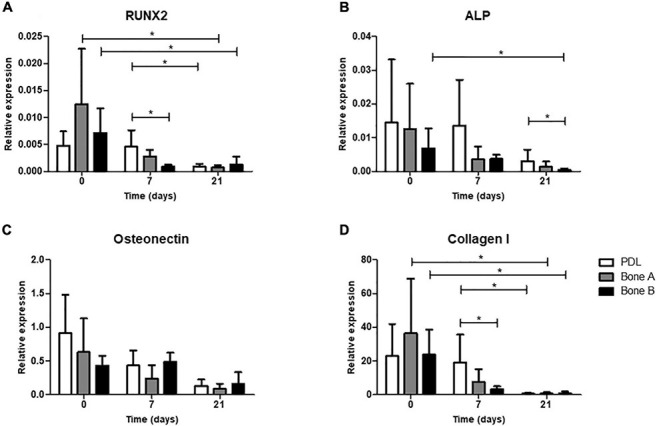
Osteogenic gene expression at day 0 (without osteogenic medium) and after 7 and 21 days of culturing under osteogenic condition. **(A)** Gene expression of early marker RUNX2, **(B)** Alkaline phosphatase, **(C)** bone matrix protein Osteonectin and **(D)** bone matrix marker Collagen I was assessed for PDL, bone A and bone B. Expression is depicted relative to housekeeping gene Beta-2-microglobulin. Note the difference in scale of the y-axes. Means ± SD are shown (*n* = 5). *p < 0.05 (Friedman test with Dunn’s multiple comparison *post hoc* test).

As a fourth and final assessment of osteogenesis, expression of several osteogenic genes was studied for PDL, bone A, and bone B. In order to get an impression on original osteogenic capacity before osteogenic conditions were applied, day 0 was sampled and compared with expression on days 7 and 21 (Figure 7). Cells from PDL, bone A, and B already showed properties of osteogenic cells at day 0, before cells were cultured under osteogenic conditions. For RUNX2, ALP, Osteonectin, and Collagen I a decrease in relative expression seemed to occur in the presence of osteogenic medium (day 7) and further decreased over time (day 21) for the 3 cell cultures. RUNX2 (Figure 7A) and Collagen I (Figure 7D) showed a similar pattern of expression. For both genes, the expression of bone A and bone B at day 0 was significant higher than at day 21 (*p* < 0.05), while PDL showed a higher expression at day 7 compared to day 21 (*p* < 0.05). PDL showed statistically significant more expression for RUNX2 and Collagen I compared to bone B at day 7 (*p* < 0.05). Despite the similar pattern, the relative expression of collagen I was much higher than RUNX2. Collagen I was also much higher expressed than ALP and Osteonectin. Bone B showed significantly lower expression of ALP at day 21 compared to day 0 (*p* < 0.05) (Figure 7B). At day 21, ALP was significant higher expressed by PDL compared to bone B (*p* < 0.05). For Osteonectin, no significant differences were found between the time points nor between the 3 cell cultures (Figure 7C). All 3 cell cultures showed very low expression for Sclerostin and almost no expression for late osteogenic marker DMP1 (data are not shown).

### Osteoclastogenesis Assays

Having established the osteogenic differences between the PDL and alveolar bone, their capacity to induce the formation of osteoclast-like cells by signaling to PBMCs was studied, as well as the induction of expression of osteoclastogenesis and osteoclast-related genes.

#### PDL Induces More Osteoclasts Than Alveolar Bone Derived Cells

**FIGURE 8:**
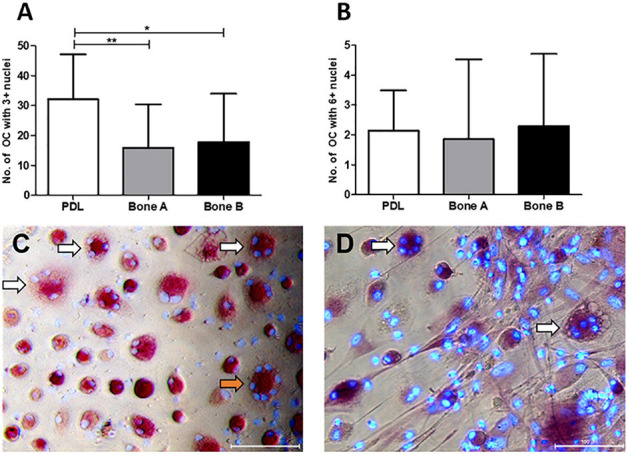
Osteoclasts at day 21. Cells from PDL, bone A and bone B were co-cultured with PBMCs as a source of osteoclast precursors. Quantification of osteoclasts was performed after staining for TRAcP activity and after staining nuclei with DAPI. Cells with at least 3 nuclei per 5 standardized fields per well were quantified. **(A)** Number of osteoclasts with ≥ 3 nulcei. PDL induced a significantly higher number of osteoclasts compared to bone A and B. **(B)** Number of osteoclasts with ≥ 6 nuclei without significant differences between the 3 cell cultures. Note the difference in scale of the y-axes between the graphs. Bars represent means ± SD. *p < 0.05; **p < 0.01 (One-way ANOVA (repeated measures) with a Tukey’s multiple comparison *post hoc* test). Microscopic images of osteoclasts with 3–5 nuclei (white arrows) and 7 nuclei (orange arrow) induced by **(C)** PDL and **(D)** bone A. Panel **(D)** also shows fibroblastic appearances of cells. Bar = 100 μm.

The number of osteoclast-like cells (further referred to as osteoclasts) induced by PDL, bone A and bone B was determined at day 21 from 7 patients (Figure 8A). Only cells with at least three nuclei were counted. Osteoclast formation took place in all cultures. PDL induced approximately two times more osteoclasts than bone A (*p* < 0.05) and bone B (*p* < 0.01). No significant difference between the number of osteoclasts in bone A and B bone was found. Figure 8B shows the category of osteoclasts with at least six nuclei. Only few of these cells were observed and no significant differences were found between the three cell cultures. Examples of clearly distinguishable osteoclasts with a microscope are shown in Figures 8C,D. Fibroblastic appearances of cells, as illustrated in Figure 8D, were mainly visible on images taken from Bone A and B.

#### qPCR Analysis of Osteoclastogenesis and Osteoclast Genes

**FIGURE 9:**
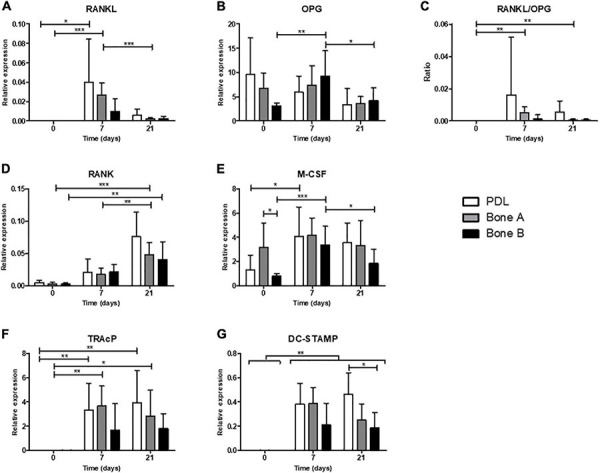
Osteoclastogenesis and osteoclast genes. Gene expression was assessed in absence of PBMCs (day 0) and after co-culturing with PBMCs (day 7 and 21) for PDL, bone A and bone B. Gene expression of **(A)** RANKL, **(B)** OPG, **(C)** RANKL/OPG ratio, **(D)** RANK, **(E)** M-CSF, **(F)** TRAcP, and **(G)** DC-STAMP are shown relative to housekeeping gene HPRT. Note the difference in scale of the y-axes. Means ± SD are shown (*n* = 7). *p < 0.05; **p < 0.01; ***p < 0.001 [One-way ANOVA (repeated measures) with a Tukey’s multiple comparison *post hoc* test].

Relative gene expression for markers of osteoclastogenesis (RANKL, OPG, RANK, and M-CSF) and for osteoclasts (later markers TRAcP and DC-STAMP) in absence of PBMCs (day 0) and after co-culturing with PBMCs (day 7 and 21) is shown in Figure 9.

#### Osteoclastogenesis Genes

Receptor activator of nuclear factor kappa-ligand expression was highly induced when co-cultured with PMBCs (days 7 and 21, Figure 9A). RANKL was higher on day 7 compared to day 21 in PDL and bone A, demonstrating its function in the (early) formation of osteoclasts. OPG was relatively highly expressed compared to the other markers. For bone B significant differences were found in expression of OPG between time points, with an increase at day 7 (*p* < 0.01) and a decrease at day 21 (*p* < 0.05) (Figure 9B). No differences over time were found for PDL and bone A. The RANKL/OPG ratio was increased at day 7 and 21 compared to day 0 (*p* < 0.01) for bone A (Figure 9C). Expression of RANK for bone A was significantly higher at the end of the co-culture period compared to day 0 (*p* < 0.001) and 7 (*p* < 0.01) (Figure 9D). Bone B also showed significantly higher expression of RANK at day 21 compared to day 7 (*p* < 0.01). Bone B expressed significantly higher levels of M-CSF after 7 days (p < 0.001) of co-culturing and subsequently decreased at day 21 (*p* < 0.05) (Figure 9E). M-CSF expression for PDL was significantly induced after co-culturing, without differences between day 7 and 21. In absence of PMBCs (day 0), bone A showed significant higher levels of M-CSF compared to bone B (*p* < 0.05). This finding was the only significant difference between cell cultures for all markers of osteoclastogenesis.

#### Osteoclast Genes TRAcP and DC-STAMP Are Highly Induced When Co-cultured With PBMCs

When co-cultured with PBMCs, the expression of TRAcP was highly induced for PDL and Bone A at day 7 (*p* < 0.05) and remained at the same level at the end of the co-culture period (Figure 9F). Bone B showed a similar pattern, but this did not reach significance. All 3 cell cultures showed highly induced expression of DC-STAMP after 7 days and 21 days of co-culturing with PMBCs compared to day 0 (*p* < 0.01) (Figure 9G). Co-cultures of PBMCs with PDL expressed higher levels of DC-STAMP compared to bone B at day 21, partially in line with the observed differences in osteoclast formation. No other differences between the cell cultures for DC-STAMP and TRAcP were found.

## Discussion

We compared the biological character of human alveolar bone and PDL cells in terms of osteogenesis and osteoclastogenesis. To the best of our knowledge, this is the first time that this topic has been investigated with the inclusion of a significant number of pairs of PDL and alveolar bone cells. Our main findings indicate that the bone remodeling capacity is slightly in favor of PDL cells. PDL cells primarily showed more mineral deposition in six out of seven cases, as qualitatively shown by Alizarin red staining, and induce approximately twice as many osteoclasts as compared to alveolar bone cells. However, when quantified by ALP activity, calcium concentration and gene expression and the expression of osteoclast(ogenesis) genes, PDL cells are generally not different from alveolar bone cells. Our study shows that alveolar bone cells also contribute to bone remodeling, since both osteogenesis and osteoclastogenesis were induced by alveolar bone cells. The bone outgrowths, bone A (early outgrowth) and B (later outgrowth, representing cells that detach later from bone chips), barely show differences in bone remodeling. Our hypothesis, stating that alveolar bone derived cells had a higher bone remodeling capacity than PDL, is rejected based on these findings.

Although the PDL and alveolar bone are two different anatomical structures, their cell cultures look similar, showing an elongated, fibroblastic-like appearance. Also the two bone outgrowths (bone A and B) are morphologically indistinguishable. These cells were previously shown to have various stem cell characteristics, such as the capacity to differentiate in various mesenchymal lineages including osteoblasts (Nagatomo et al., 2006; Zhang et al., 2012; Straka et al., 2016). Indeed, it was shown by Nomura et al. (2012) and by Sanchez-Duffhues et al. (2019) that PDL fibroblasts are an ideal source for making induced pluripotent stem cells. The morphology of alveolar bone derived cells *in vitro* is also in agreement with the study by Kelder et al. (2020), in which alveolar bone was compared to long bone cells. In that study, long bone cells presented a more cuboidal appearance, similar to the morphology of osteoblasts. This difference in morphology compared to long bone, its similarity to cell cultures from PDL and its contribution to both osteogenesis and osteoclastogenesis assays as shown in the present study, might indicate that the cell cultures from alveolar bone also have certain stem cell characteristics. Indeed, with the qPCRs we have established the stem cell nature of the three cell cultures, with CD73 expression that was not different between the cell lineages, nor were differences observed with mesenchymal markers Vimentin, Periostin, PLAP-1 and FAPα with only slight differences in Scleraxis expression. Of particular interest is the protein Periostin, which is expressed highest in PDL fibroblasts at the basal part of the tooth, away from the gingiva. Here, it is associated with focal adhesions as well as Sharpey’s fibers (Wen et al., 2010).

To analyze the osteogenic differentiation of PDL and alveolar bone derived cells, we studied the ALP activity, calcium concentration and gene expression levels of specific osteogenic genes under osteogenic conditions. ALP is an important enzyme for bone matrix mineralization by promoting the osteogenic differentiation of osteoprogenitor cells (Nakamura et al., 2020). Calcium deposition by osteoblasts is the final process of mineralization and is therefore only assessed at day 14 and 21 (de Vries et al., 2018). Cell proliferation was similar between the cultures and progressed until confluence of the cultures, as typically seen in fibroblast cultures. For both the ALP activity as the calcium concentration no statistically significant differences were found between PDL, bone A and bone B, albeit that the PDL values seemed somewhat higher. ALP activity and calcium concentration increased over time. This indicates that the cells become more differentiated over time. Ruppeka-Rupeika et al. (2018) found a significantly higher ALP activity of PDL compared to alveolar bone at day 14 in a case study. The difference with the present study might be due to the sample size and health of the patient(s), since they only included cells from one healthy donor with a less well-defined piece of bone that was attached to the tooth, whereas our study included samples from seven patients, but with compromised health.

In the present study, we investigated the expression of typical osteogenic markers RUNX2, ALP, Osteonectin and Collagen I, Sclerostin, and DMP1. Sclerostin is a late osteogenic marker, since it is exclusively secreted by osteocytes (Pietrzyk et al., 2017), which are entrapped osteoblasts within the matrix they secrete. All three cell cultures showed very low or absent expression of Sclerostin, indicating that overall, these cells did not differentiate into late osteogenic lineage. Likewise, PDL and alveolar bone cells hardly expressed late osteogenic marker DMP1, an important marker for bone mineralization. Low expression levels of DMP1 were also previously found (Ruppeka-Rupeika et al., 2018; Kelder et al., 2020). This might indicate that under these circumstances, cells do not differentiate fully until the late osteoblast/osteocyte stage. Interestingly, the expression at day 0 of RUNX2, ALP, Osteonectin and Collagen I in all three cell types showed an intrinsic osteogenic potential at the start of the experiment. When the cells were exposed to osteogenic medium, a pattern of decrease in expression is shown over time, but only with some significant differences between time points for RUNX2, ALP, and Collagen I. This is an unexpected finding, since most literature show increases over time when using osteogenic medium. Our results indicate that osteogenesis was facilitated at the start and that even the decreased expression over time resulted in further propagation of osteogenesis, resulting in increased ALP activity, Alizarin red staining and calcium deposition. The only significant differences in osteogenic markers between the cell cultures was a higher expression for PDL cells compared to bone B at day 7 for RUNX2 and Collagen I and at day 21 for ALP. These minor differences are in accordance to Liu et al. (2012), who showed that simvastatin, a cholesterol lowering drug, affected the osteogenic differentiation of both human alveolar osteoblasts and PDL cells *in vitro*, without a clear difference between the two cell cultures. We found no significant differences in osteogenic gene expression between bone A and B. This indicates that RNA and protein are not always correlated. RUNX2 and Collagen I had a similar pattern of expression. The decrease in expression of RUNX2, a key transcription factor associated with osteoblast differentiation (Komori, 2006), might indicate that the cells were already in an early stage prepared to bone-forming cells. Collagen I was much higher expressed than the other markers, showing its importance in osteogenesis as it encodes for type I collagen, the most abundant bone matrix protein (Sodek and McKee, 2000).

Alizarin red staining showed a certain degree of variability between cell cultures from different patients were found. For the majority of the patients, PDL showed stronger staining compared to alveolar bone cells. Cells obtained from 1 patient showed the opposite, where a strong staining was seen for bone A and B and absence of staining for PDL cells. Notably, PDL cells from this patient also showed the lowest values in the included quantitative osteogenesis assays compared to alveolar bone cells. No difference in mineralization was shown between bone A and bone B, but some more variety was seen for alveolar bone cells between patients. This patient strikingly used a lot of medication (see Supplementary Table 1), it could be possible that the mix of medication specifically interferes with the mineralization capacity of PDL cells. Another explanation could be that the outgrowth of this PDL contained mainly stem cells that were osteogenesis negative. Recently, Ueda et al. (2021) described cell clones isolated from PDL that were highly osteogenic versus clones that were very lowly osteogenic.

None of the osteogenesis assays showed a difference between bone A and bone B. Later outgrowth of alveolar bone cells (bone B) do not differ in osteogenic capacity from the earlier outgrowth, that represent cells that detach from bone earlier (bone A).

PDL cells induced significantly, approximately two times more osteoclasts compared to bone A and bone B, and no differences in osteoclastogenesis were observed between these two bone outgrowths. Osteoclasts with 6 or more nuclei were a rare finding.

The higher gene expression of M-CSF for bone A compared to bone B in the absence of PBMCs (day 0) was the only significant difference between cell cultures for the investigated markers of osteoclastogenesis (RANKL, OPG, RANK, and M-CSF). M-CSF stimulates the hematopoietic stem cell to differentiate into the osteoclast progenitor cell. The signaling pathway of RANK, expressed on the membrane of osteoclast progenitors, and its ligand, RANKL, play a major role in controlling osteoclastogenesis (Boyce, 2013). RANKL was highly expressed once the cells were co-cultured with PBMCs, which is in accordance with the study of Bloemen et al. (2010), who found that co-culture dramatically increases expression of RANKL compared to mono-cultures of PBMCs or PDL cells. *In vivo*, it was shown that RANKL is expressed more in alveolar bone cells than in PDL cells, consistent with its role in osteoclast formation where orthodontic tooth movement takes place (Shoji-Matsunaga et al., 2017). OPG, an osteoclastogenesis-inhibitor by preventing RANKL-RANK interaction, was much higher expressed than RANKL. Nevertheless, osteoclasts cells were still formed. This is confirmed in several other studies (Kanzaki et al., 2001; Bloemen et al., 2010; Kook et al., 2011). A possible explanation for this phenomenon could be that the tight cell-cell contact between PDL cells and PBMCs creates a micro-environment which is favorable for RANKL-RANK binding and thereby preventing the inhibitory role of OPG with RANKL (Bloemen et al., 2010). In addition to the osteoclastogenesis markers, expression of the osteoclast markers TRAcP and DC-STAMP was measured. TRAcP is a histochemical marker of osteoclasts but also has important functions in the skeleton and immune system (Hayman, 2008), while DC-STAMP is essential for the cell-to-cell fusion of precursor cells to form the mature multinucleated osteoclasts (Chiu and Ritchlin, 2016). As expected, both markers were highly expressed when co-cultured with PMBCs (day 7 compared to day 0). DC-STAMP expression was significantly higher for PDL-PBMC co-cultures compared to bone B—PBMC co-cultures, being the only significant differences between cell cultures for these late markers. This difference is in line with our other finding that PDL induced more osteoclasts compared to alveolar bone cells. In the present study, the inability of osteoclasts to resorb bone, when originating from fibroblast-PBMC co-cultures (de Vries et al., 2006), was not assessed since this was out of scope of our research aim. Next to differences in the dynamics of osteoclastic activity (Delaisse et al., 2021), our study proposes that also differences in osteoclastogenesis may contribute to site-tailored degree of osteoclast formation.

Bearing in mind all limitations of *in vitro* studies, for instance culturing on plastic and isolating one particular cell type, a strength of this study is that paired comparisons were performed for all results, since alveolar bone cells and PDL cells were obtained from the same patient. In addition, this is the first study that investigated the bone remodeling capacity of alveolar bone and PDL samples from seven different patients. The alveolar bone particles were obtained as part of the preparation phase for a better fit of the immediately placed removable (partial) denture. All patients in the current study had compromised general and oral health. This might have been the reason for the inter-individual variability in bone remodeling capacity. The poor oral health could also mean the inflammation has changed the phenotype of the cells. So far, almost all *in vitro* studies have used the roots of third molars as a source for human PDL cells (Basdra and Komposch, 1997; Seo et al., 2004; Zhang et al., 2012; de Vries et al., 2018), since these molars are frequently extracted while their periodontal attachment apparatus is still intact, which makes them readily available and suitable sources to retrieve cells from. In the present study, the donor teeth were extracted because of caries and/or periodontal problems and were often surrounded by inflamed tissues. Human PDL stem cells from inflamed tissues keep their regenerative potential as compared to PDL cells from healthy tissues (Park et al., 2011), but also show a different immune response by less suppression of T-cell differentiation and proliferation (Liu et al., 2012) and a more active interaction with the periodontal pathogen Porphyromonas gingivalis (Scheres et al., 2011).

Re-establishing the original surroundings of the tooth by regeneration of the lost periodontium is the ultimate goal in treating periodontitis (Hägi et al., 2014). Isolated human PDL stem cells *in vitro* has been successfully used in animal models for the purpose of regeneration of Seo et al. (2004). In most of the cases, regenerative materials are exposed to inflammatory circumstances when used intraorally. The present study included cells that originated from inflamed tissues, but the assays were not performed under inflammatory circumstances. PDL stem cells exhibit weaker osteogenic differentiation compared to bone marrow mesenchymal stem cells under an inflammatory *in vitro* environment (Zhang et al., 2014). Compared to bone marrow cells from the iliac crest, cells from the maxilla and mandible proliferate more rapidly and express higher ALP and calcium *in vitro* (Akintoye et al., 2006). Furthermore, stem cells from the jawbone might be suitable candidates for regeneration of alveolar bone (Matsubara et al., 2005; Nishimura et al., 2012). It could therefore be of utmost interest to compare the bone remodeling capacity of alveolar bone cells with PDL cells in an inflammatory environment.

The alveolar bone chips were cut into several small fragments, increasing its available cultivable surfaces from multiple directions. The PDL faces cortical bone, whereas bone A and bone B are retrieved from both cortical as well as trabecular bone. This could be one of the reasons for the differences in the present study between PDL and alveolar bone cells. Our results show that for future research, it is not needed to include several bone outgrowths, since the two different outgrowths (bone A and bone B) barely showed differences in bone remodeling capacity.

Age of the patient and the location of the jaw from which the samples were obtained were heterogeneous and could rightfully be seen as a possible limitation of the study. We were able to successfully isolate PDL cells from patients of different ages (a wide range of 21–75 years), as was previously shown by Zhang et al. (2012). In the present study, 6 pairs of samples (the extracted tooth and alveolar bone) were obtained from the lower jaw and only one sample originated from the upper jaw (Table 2). It is also widely known that the bone density between the upper and lower jaw is markedly different, with the upper jaw being less dense and strong, but more vascularized than the lower jaw (Oliveira et al., 2021). These aspects might be important in bone remodeling research and could be elucidated in future studies.

## Concluding Remarks

Our results suggest that PDL cells are slightly more active in bone remodeling processes. Cells derived from human alveolar bone also possess the capacity to mineralize *in vitro* and to provide signals for the formation of osteoclasts, but this could be to a different extent. The present study furthermore shows that human alveolar bone derived cells can be used as an *in vitro* model to study bone remodeling capacity, creating possibilities for a shared focus between PDL cells and alveolar bone cells in future bone remodeling related research in dentistry.

## Data Availability Statement

The raw data supporting the conclusions of this article will be made available by the authors, without undue reservation.

## Ethics Statement

The studies involving human participants were reviewed and approved by the Research Ethics Committee of OLVG (protocol-ID: WO 17.194). The patients/participants provided their written informed consent to participate in this study.

## Author Contributions

RL-K: design of the study, conducting the experiments, and writing the manuscript. MG: collection of patient material and assistance with medical ethical approval. JH and TS: expert assistance with the laboratory experiments. TdV: design of the study, conducting the experiments, and editing all versions of the manuscript. All authors contributed to the article and approved the submitted version.

## Conflict of Interest

The authors declare that the research was conducted in the absence of any commercial or financial relationships that could be construed as a potential conflict of interest.

## Publisher’s Note

All claims expressed in this article are solely those of the authors and do not necessarily represent those of their affiliated organizations, or those of the publisher, the editors and the reviewers. Any product that may be evaluated in this article, or claim that may be made by its manufacturer, is not guaranteed or endorsed by the publisher.
