# Supplementary material for: Although Anatomically Micrometers Apart: Human Periodontal Ligament Cells Are Slightly More Active in Bone Remodeling Than Alveolar Bone Derived Cells
Source: Front Cell Dev Biol. 2021 Sep 20;9:709408. doi: 10.3389/fcell.2021.709408 (PMC8488427; doi:10.3389/fcell.2021.709408)
Supplement: Supplementary file 1 [file Data_Sheet_1.PDF]

Supplementary Table 1

Supplementary table 1 | General and health related information about the patients

**Patient**	**Age**	**Sex**	**Ethnicity**	**General health**	**Medication**	**Smoking**
**1**	67	M	Dutch	Diabetes mellitus<br>Hypertension<br>Hypercholestero-lemia	Alirocumab<br>Clopidogrel<br>Ezetrol<br>Fluoxetine<br>Metformin<br>Nifidipine<br>Ranitidine<br>Seloken	Quit 15 years ago (30 pack years*)
**2**	60	F	Turkish	Asthma<br>Diabetes mellitus<br>Hypertension<br>Hypercholestero-lemia<br>Hyperlipidemia	Ascal<br>Dapagliflozin<br>Glimeperide<br>Labetalol<br>Losartan<br>Metformin<br>Pantoprazole<br>Salbutamol<br>Seretide	No
**3**	21	F	Dutch	Leber's Hereditary Optic Neuropathy (LHON)	Contraceptive pill	10 cig. (3 pack years*) and 1 gm cannabis per day
**4**	41	F	Moroccan	Diabetes mellitus<br>Overweight	No	No
**5**	57	F	Dutch	Stroke (10 years ago)	Ascal<br>Persantine<br>Pantoprazole<br>Simvastatin	No
**6**	75	M	Dutch		Cetirizine<br>Cromoglicic acid	No
**7**	37	M	Dutch	Depression	Methylphenidate<br>Omeprazole<br>Venlafaxine	5 cig. per day (5 pack years*)<br>cannabis
Mean age	51 (± 18.9)					

(Mean) age in years (SD). M: male; F: female.
Pack year = twenty cigarettes smoked every day for one year as a measure for the smoking history
Cig. = cigarettes
